# Supplementary material for: The evolutionary origin of naturally occurring intermolecular Diels-Alderases from Morus alba
Source: Nat Commun. 2024 Mar 20;15:2492. doi: 10.1038/s41467-024-46845-0 (PMC10954736; doi:10.1038/s41467-024-46845-0)
Supplement: Supplementary file 1 — Supplementary Information [file 41467_2024_46845_MOESM1_ESM.pdf]

Supplementary Information

The evolutionary origin of naturally occurring intermolecular Diels-Alderases from *Morus alba*

Qi Ding[1,2†], Nianxin Guo[2,3,4†], Lei Gao[2*], Michelle McKee[5], Dongshan Wu[2], Jun Yang[2,3], Junping Fan[2], Jing-Ke Weng[5,6,7], Xiaoguang Lei[2,3,4,8*]

Supplementary method 1. Chemical synthesis: General & The syntheses of compound 6.

Chemical synthesis of compound 6.

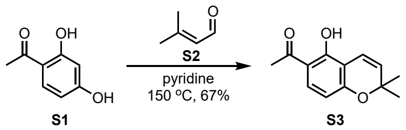

S2 (289.4 μL, 3.0 mmol) was added to a solution of **S1** (456 mg，3.0 mmol) in pyridine (0.5 mL). The reaction mixture was stirred at 150 °C for 4 hours, then **S2** (289.4 μL, 3.0 mmol) was added again and the reaction mixture was stirred at 150 °C for 6 hours. After concentrated in vacuum, the mixture was purified by silica column chromatography (petroleum ether/ethyl acetate, 50/1) to furnish the desired product **S3** (436.5 mg，67%) as a white solid.

m.p. = 97-99 °C；

^{1}H NMR (400 MHz, $CDCl_3$) δ 12.97 (s, 1H), 7.51 (d, *J* = 8.8 Hz, 1H), 6.71 (d, *J* = 10.1 Hz, 1H), 6.33 (d, *J* = 8.8 Hz, 1H), 5.58 (d, *J* = 10.0 Hz, 1H), 2.53 (s, 3H), 1.45 (s, 7H);

^{13}C NMR (100 MHz, $CDCl_3$) δ 202.7, 159.7, 159.6, 131.6, 128.2, 115.8, 113.9, 109.2, 108.3, 77.7, 28.3, 26.2;

IR (neat) ν_{max} 2976, 1620, 1486, 1363, 1330, 1270, 1211, 1111, 1072 cm^{-1};

HRMS (ESI) $[M+H]^+$ calcd. for $C_{13}H_{15}O_3^+$219.1016, found 219.1020.

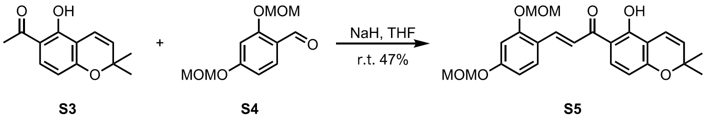

To a solution of **S3** (14 mg, 0.064 mmol) and **S4** (16 mg, 0.071 mmol) in THF (1 mL) was added NaH (5.6 mg, 0.14 mmol) at 0 °C. The reaction mixture was slowly warmed to room temperature. After stirring for 24 hours, the reaction mixture was quenched by water, neutralized to pH = 5 by addition of 2M HCl aqueous solution and extracted with ethyl acetate (10 mL *3). The organic layers were washed with brine (5 mL), dried over anhydrous sodium sulfate. After concentrated in vacuum, the mixture was purified by silica column chromatography (petroleum ether/ethyl acetate, 10/1) to furnish the desired product **S5** (13.0 mg, 47%) as a yellow solid.

m.p. = 84-86 °C;

^{1}H NMR (400 MHz, $CDCl_3$) δ 13.86 (s, 1H), 8.18 (d, *J* = 15.6 Hz, 1H), 7.71 (d, *J* = 8.9 Hz, 1H), 7.60 (d, *J* = 8.7 Hz, 1H), 7.56 (d, *J* = 15.6 Hz, 1H), 6.86 (d, *J* = 2.3 Hz, 1H), 6.81 – 6.65 (m, 2H), 6.37 (d, *J* = 8.8 Hz, 1H), 5.59 (d, *J* = 10.0 Hz, 1H), 5.28 (s, 2H), 5.20 (s, 2H), 3.52 (s, 3H), 3.49 (s, 3H), 1.46 (s, 6H);

^{13}C NMR (100 MHz, $CDCl_3$) δ 192.4, 160.9, 160.5, 159.6, 157.9, 139.4, 130.5, 129.90, 128.0, 118.8, 118.5, 116.0, 114.2, 109.4, 109.4, 108.1, 103.3, 94.7, 94.3, 77.7, 56.4, 56.3, 28.3;

IR (neat) ν_{max} 2929, 1627, 1604, 1579, 1557, 1482, 1356, 1292, 1255 cm^{-1};

HRMS (ESI) $[M+H]^+$ calcd. for $C_{24}H_{27}O_7^+$ 427.1751, found 427.1750.

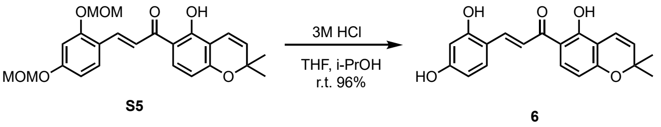

To a solution of **S5** (19.0 mg，0.045 mmol) in THF (0.8 mL) and i-PrOH (0.8 mL), 3M HCl (0.4 mL) was added slowly at 0 °C. The reaction mixture was slowly warmed to room temperature. After stirring for 32 hours, the reaction mixture was quenched by water and extracted with ethyl acetate (10 mL *3). The organic layers were dried over anhydrous sodium sulfate, concentrated in vacuum and purified by preparative TLC to furnish the desired compound **6** (16.3 mg, 96%) as a yellow solid.

^{1}H NMR (400 MHz, acetone-*d*6) δ 14.24 (s, 1H), 9.09 (d, J = 123.9 Hz, 2H), 8.25 (d, J = 15.4 Hz, 1H), 7.98 (d, J = 8.9 Hz, 1H), 7.80 (d, J = 15.4 Hz, 1H), 7.73 (d, J = 8.6 Hz, 1H), 6.71 (d, J = 10.0 Hz, 1H), 6.52 (d, J = 2.2 Hz, 1H), 6.47 (dd, J = 8.6, 2.2 Hz, 1H), 6.37 (d, J = 8.8 Hz, 1H), 5.72 (d, J = 10.1 Hz, 1H), 1.45 (s, 6H);

^{13}C NMR (100 MHz, acetone-*d*6) δ 192.7, 161.6, 160.8, 159.3, 159.2, 140.5, 131.1, 131.0, 128.30, 116.3, 115.5, 114.3, 114.1, 109.1, 108.4, 107.9, 102.7, 77.5, 27.6;

IR (neat) ν_{max} 2926, 1612, 1579, 1482, 1363, 1297, 1239, 1116 cm^{-1};

HRMS (ESI) $[M+H]^+$ calcd. for $C_{20}H_{19}O_5^+$ 339.1227, found 339.1227.

Supplementary method 2. Enzymatic synthesis: *endo*-8 & *exo*-9.

The method for preparative HPLC.

Preparative HPLC was performed on a Waters purification system equipped with a Waters 2998 photodiode array detector and XBridge pre-C18 optimum bed density column (Waters; length, 150 mm; inner diameter, 19 mm; particle size, 5 μm) at a flow rate of 20 ml/min at 25 °C using a gradient elution of water (A) and MeCN (B). The gradient program was 30% B, 0–1 min, 30–95% B, 1–16 min, 95% B, 16–20 min.

Enzymatic synthesis of *endo*-8.

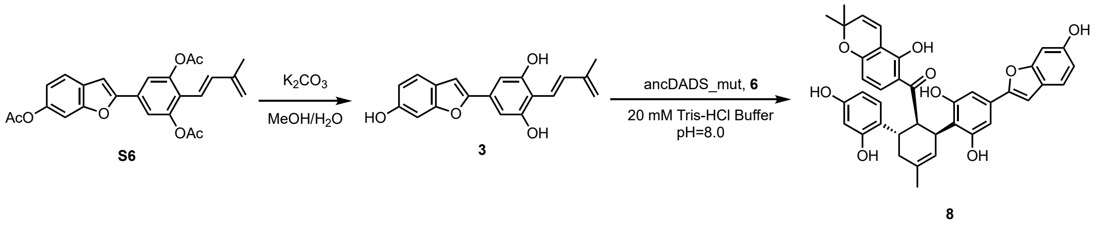

Diene precursor **S6** (8.0 mg, 0.0184 mmol) was added to 1 mL mixture solution of MeOH and H_2O ($MeOH/H_2O$ = 4:1). The reaction mixture was degassed by bubbling argon for 10 min, and then K_2CO_3 (12.0 mg, 0.0873 mmol) was added under argon atmosphere. The resulting mixture was stirred for 35 min to generate diene **3** *in situ.* Then the resulting solution was added to 50 mL reaction solution (7.35 mg, 108 nM ancDADS-mut5b in 20 mM Tris-HCl, pH = 8.0). To this mixture, dienophile (**6**, 4.4 mg dissolved in 0.13 mL DMSO, 0.013 mmol) was added. The resulting mixture was incubated at 37 °C for 43 hours. The resulting mixture was neutralized to pH =7 by addition of 50 mL saturated NH_4Cl then extracted with ethyl acetate (100 mL *3). The organic layers were dried over anhydrous sodium sulfate, concentrated in vacuum and purified by HPLC as described above to give ***endo*-8** (1.4 mg, 17%) as an off-white solid. The NMR data was consistent as previously reported[6].

^{1}H NMR (400 MHz, Acetone-*d6*) δ 12.97 (s, 1H), 8.76 (s, 1H), 8.59 – 8.33 (m, 2H), 8.08 (d, J = 10.4 Hz, 3H), 6.98 (d, J = 8.4 Hz, 1H), 6.93 (d, J = 2.6 Hz, 2H), 6.78 (d, J = 2.0 Hz, 1H), 6.77 (s, 2H), 6.58 (d, J = 10.1 Hz, 1H), 6.49 (d, J = 2.5 Hz, 1H), 6.30 (dd, J = 8.4, 2.4 Hz, 1H), 6.25 (d, J = 8.9 Hz, 1H), 5.78 (s, 1H), 4.16 (s, 1H), 2.51 (d, J = 10.3 Hz, 1H), 2.26 (s, 1H), 1.40 (s, 3H), 1.37 (s, 3H).

HRMS (ESI) calcd. for $C_{39}H_{35}O_9$ $[M+H]^+$ 647.2276, found 647.2287.

Enzymatic synthesis of *exo*-9.

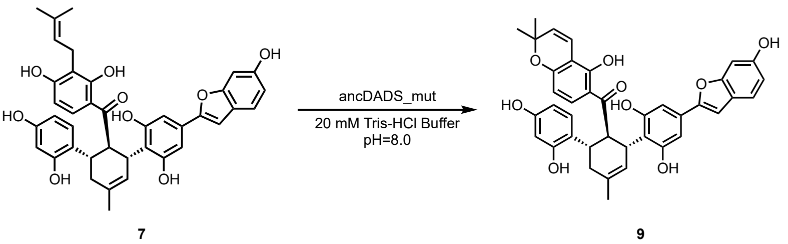

Mongolicin F **7** (3.0 mg, 0.00463 mmol) was added to 50 mL reaction solution (5.05 mg, 68 nM ancDADS-mut6 in 20 mM Tris-HCl, pH = 8.0). The resulting mixture was incubated at 37 °C for 5.5 hours. The resulting mixture was neutralized to pH =7 by addition of 50 mL saturated NH_4Cl then extracted with ethyl acetate, and purified by HPLC to give ***exo*-9** (2.7 mg, 90.3%) as an off-white solid.

^{1}H NMR (500 MHz, CD_3SOCD_3) δ 13.26 (s, 1H), 8.89 (s, 1H), 8.65 (s, 1H), 8.45 (s, 1H), 7.58 (d, J = 8.9 Hz, 1H), 7.33 (d, J = 8.3 Hz, 1H), 6.87 (d, J = 2.1 Hz, 1H), 6.77 (d, J = 0.9 Hz, 1H), 6.74 (s, 0H), 6.73 (d, J = 2.1 Hz, 0H), 6.71 (d, J = 2.1 Hz, 0H), 6.60 (s, 2H), 6.41 (d, J = 10.0 Hz, 1H), 6.14 (d, J = 2.4 Hz, 1H), 5.98 (dd, J = 8.3, 2.4 Hz, 2H), 5.53 (d, J = 10.0 Hz, 1H), 5.35 (td, J = 5.6, 4.4, 1.1 Hz, 2H), 5.24 (s, 1H), 4.85 (t, J = 10.7 Hz, 1H), 4.35 (d, J = 9.8 Hz, 1H), 3.61 – 3.48 (m, 1H), 2.19 (d, J = 1.4 Hz, 1H), 1.70 (s, 3H), 1.18 (s, 3H);

^{13}C NMR (151 MHz, CD_3SOCD_3) δ 174.3, 161.2, 158.2, 157.5, 155.9,155.6, 155.1, 153.8, 129.6, 128.6, 128.3, 124.8, 121.0, 120.8, 115.8, 115.0, 114.8, 112.3, 102.8, 101.9, 100.8, 97.4, 77.4, 31.3, 28.7, 27.9, 26.6, 25.1, 22.1, 14.0;

$[\alpha]_D^{25}$ = -105.677° (c = 0.20, in MeOH);

IR (neat) ν_{max} 3357.88, 2924.4, 2853.1, 1661.2, 1618.2, 1425.4, 1260.8, 1117.3, 1037.2 cm^{-1};

HRMS (ESI) calcd. for $C_{39}H_{35}O_9$ $[M+H]^+$ 647.2276, found 647.2273.

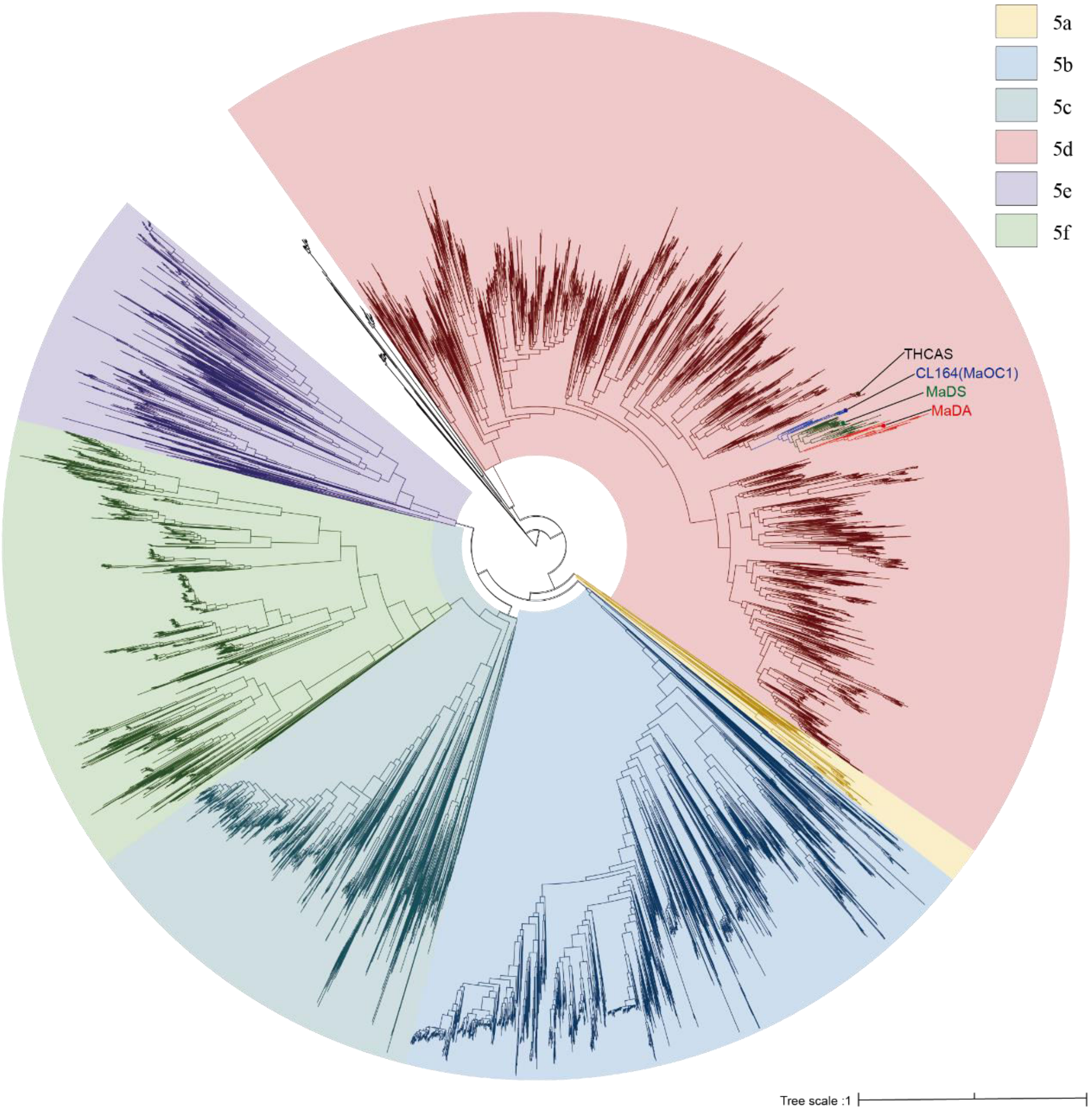

Supplementary Fig. 1| The phylogenetic tree of BBE-like enzyme family.

A phylogenetic tree of the BBE-like enzyme family was constructed using the maximum likelihood method, based on 7,576 proteins. The proteins were divided into six clades (5a-5f).

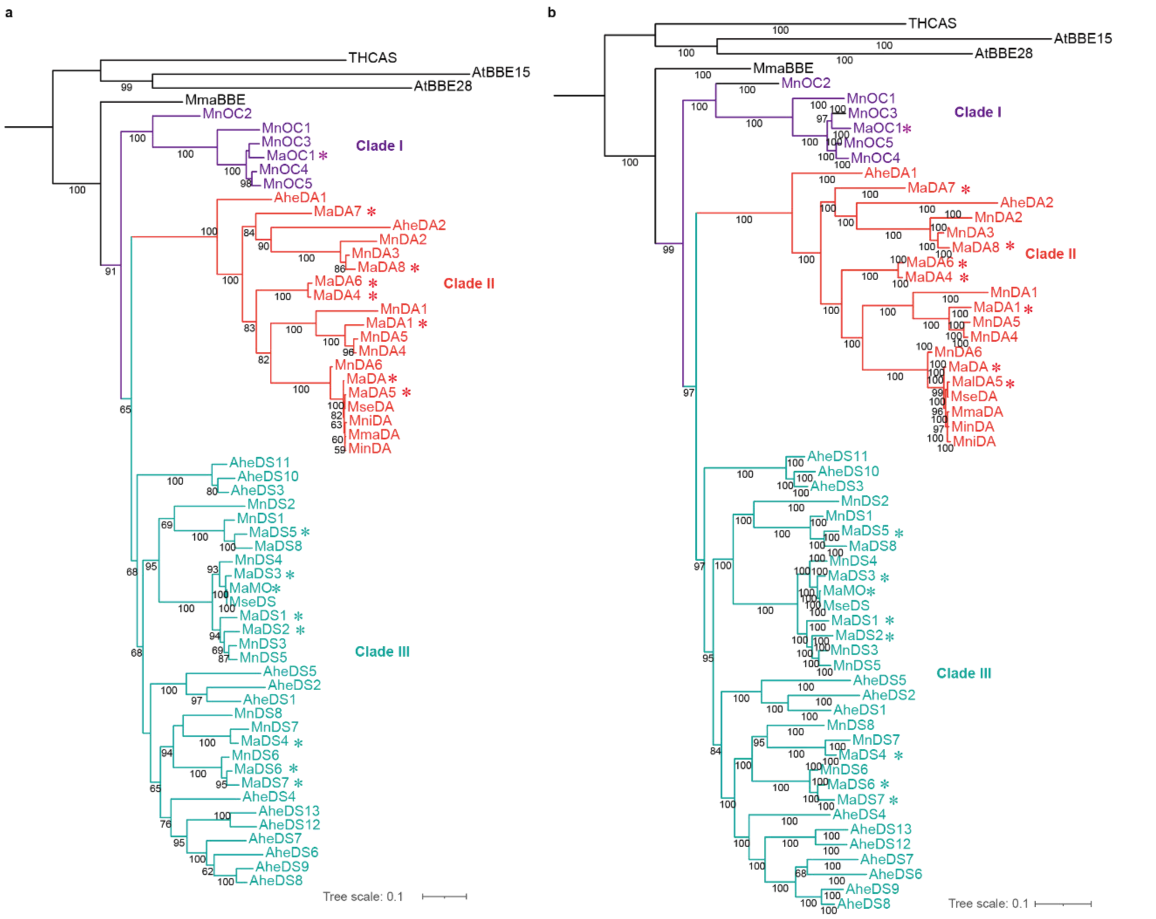

Supplementary Fig. 2| Phylogenetic tree constructed based on the maximum parsimony and Bayesian inference methods.

The phylogenetic trees constructed with Phylip (**a**) and MrBayes (**b**). The percent bootstrap values are presented for each clade with values >60%. Ahe: *Artocarpus heterophyllus*; Min: *Morus indica*; Mni: *Morus nigra*; Mse: *Morus serrata*; Mma: *Morus macroura*; Ma: *Morus alba*; Mn: *Morus notabilis.* Genes labeled with asterisks are utilized for subsequent functional validation.

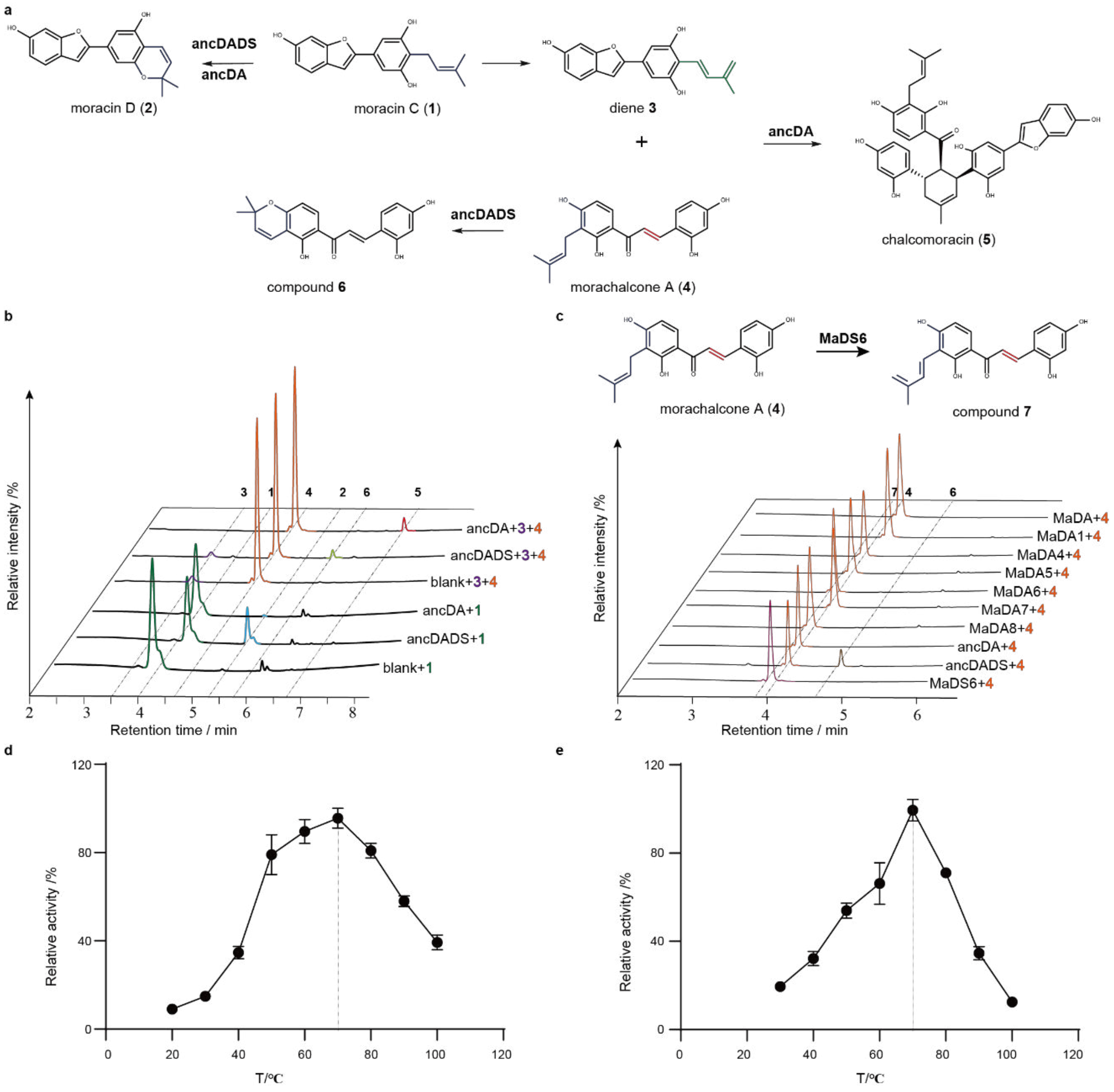

Supplementary Fig. 3| Functional characterization and optimal reaction temperatures of ancestral genes.

a, The reaction catalysed by ancDADS and ancDA. **b,** Ultra-performance liquid chromatography (UPLC) analysis of the enzymatic reactions catalysed by ancDADS and ancDA. The enzyme assay experiment was conducted with three independent replicates. **c,** UPLC analysis of the enzymatic assays of the ancestral enzymes and DAs on morachalcone A (**4**). The enzyme assay experiment was conducted with three independent replicates. Effect of temperature on ancDADS's activity toward moracin C (**d**) and morachalcone A (**e**). The enzyme assays were conducted at different temperatures (20-100 °C) for 7 minutes in 100 µL Tris-HCl buffer (20 mM, pH = 8.0) containing either moracin C (**1**) (100 µM) or morachalcone A (**4**) (100 µM) as the substrate and 5 µg ancDADS. The data are presented as mean values ± standard error (S.E.), with error bars indicating the standard deviations of three independent measurements.

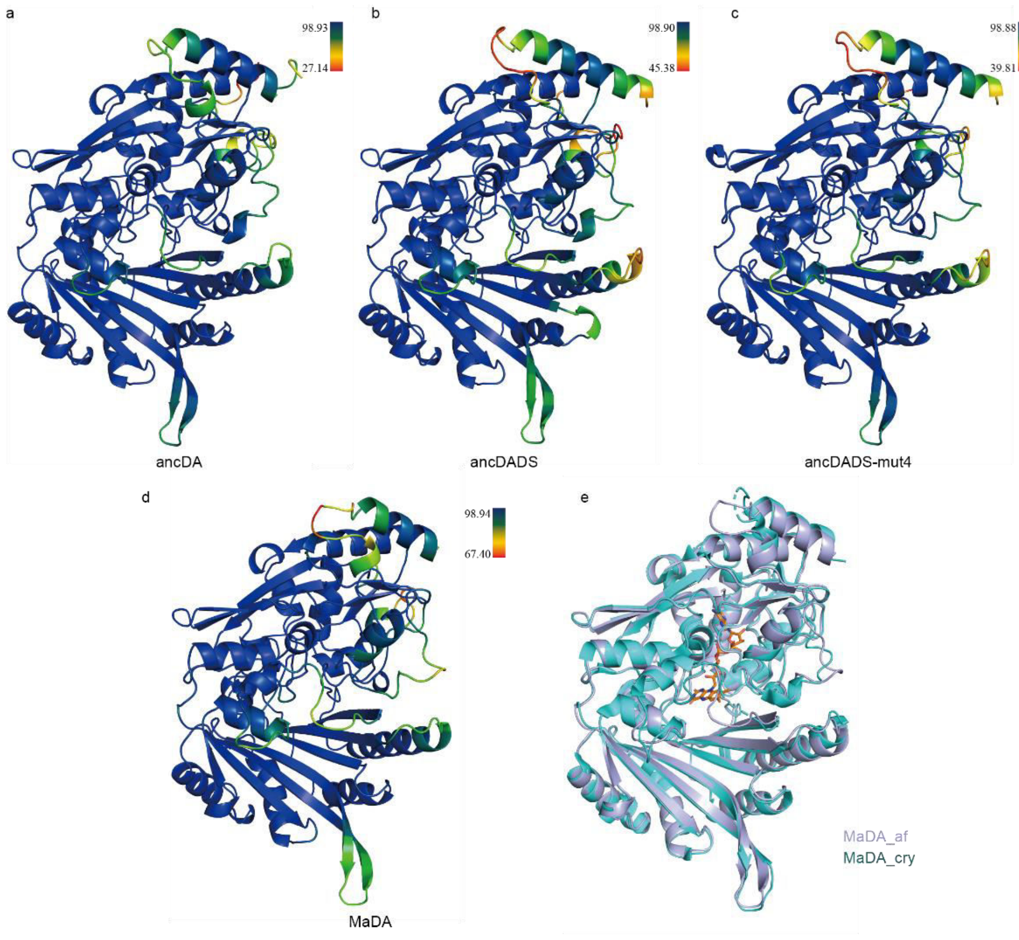

Supplementary Fig. 4| Modelled structures of the four BBE-like enzymes using AlphaFold.

a-d, Predicted structures for ancDA, ancDADS, ancDADS-mut4, and MaDA are presented. Residues are color-coded based on their per-residue confidence score (pLDDT), ranging from the minimum value to the maximum value. Lower pLDDT values indicate lower confidence, while higher values signify more confident predictions. **e,** An alignment is shown between the predicted structure (light purple) of MaDA and the crystal structure of MaDA (cyan, PDB ID 6JQH).

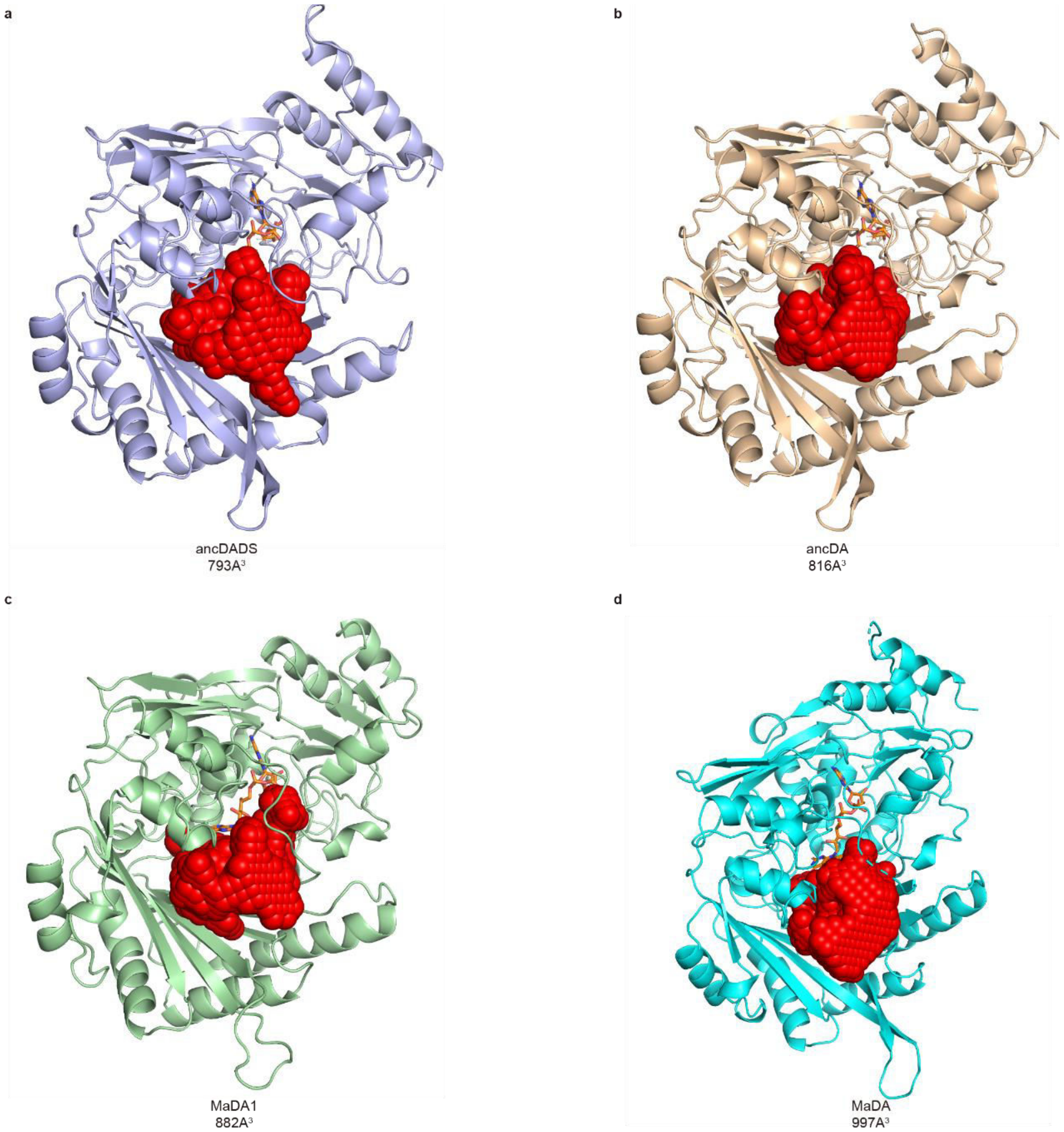

Supplementary Fig. 5| Comparing the volumes of the enzyme binding pockets.

The calculated pockets and their volumes for the ancDADS (**a**), ancDA (**b**), MaDA1 (**c**), and MaDA (**d**), respectively.

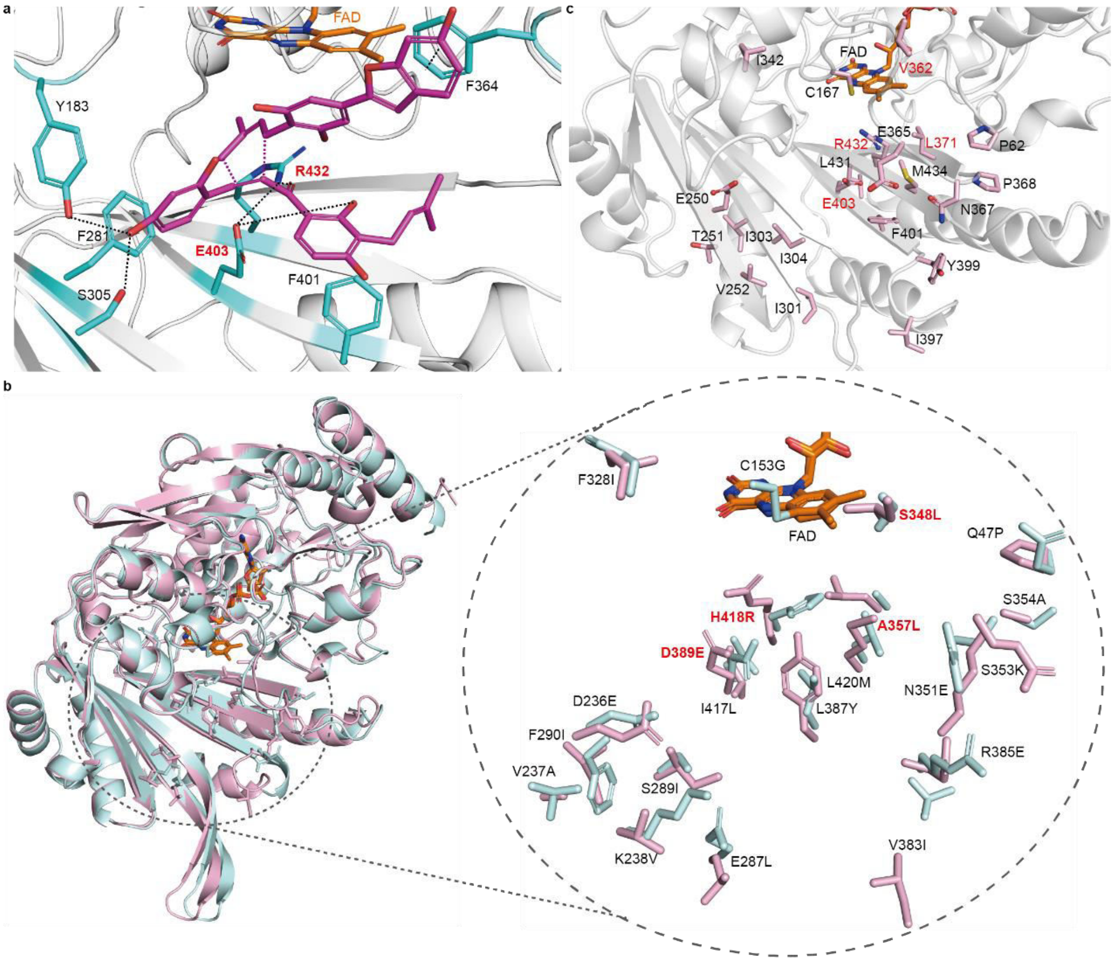

Supplementary Fig. 6| The enzyme pocket of MaDA1 and the 21 differential residues.

a, The binding model of the transitional state in MaDA1. The substrate is purple and residues that interact with the substrate are marked in cyan. **b,** The corresponding residues in the enzyme pocket of MaDA1 that are differential between ancDA and ancDADS. The corresponding residues that are evolutionally important for the emergence of D-A function are highlighted in red. **c,** The 21 differential residues in the enzyme pockets of ancDA (light pink) and ancDADS (pale cyan). Taken "H418R" as an example, this word means that the 418th residue of ancDADS is a histidine (H) while the corresponding residue in ancDA is an arginine (R). The most evolutionally important residues for the emergence of D-A function are highlighted in red.

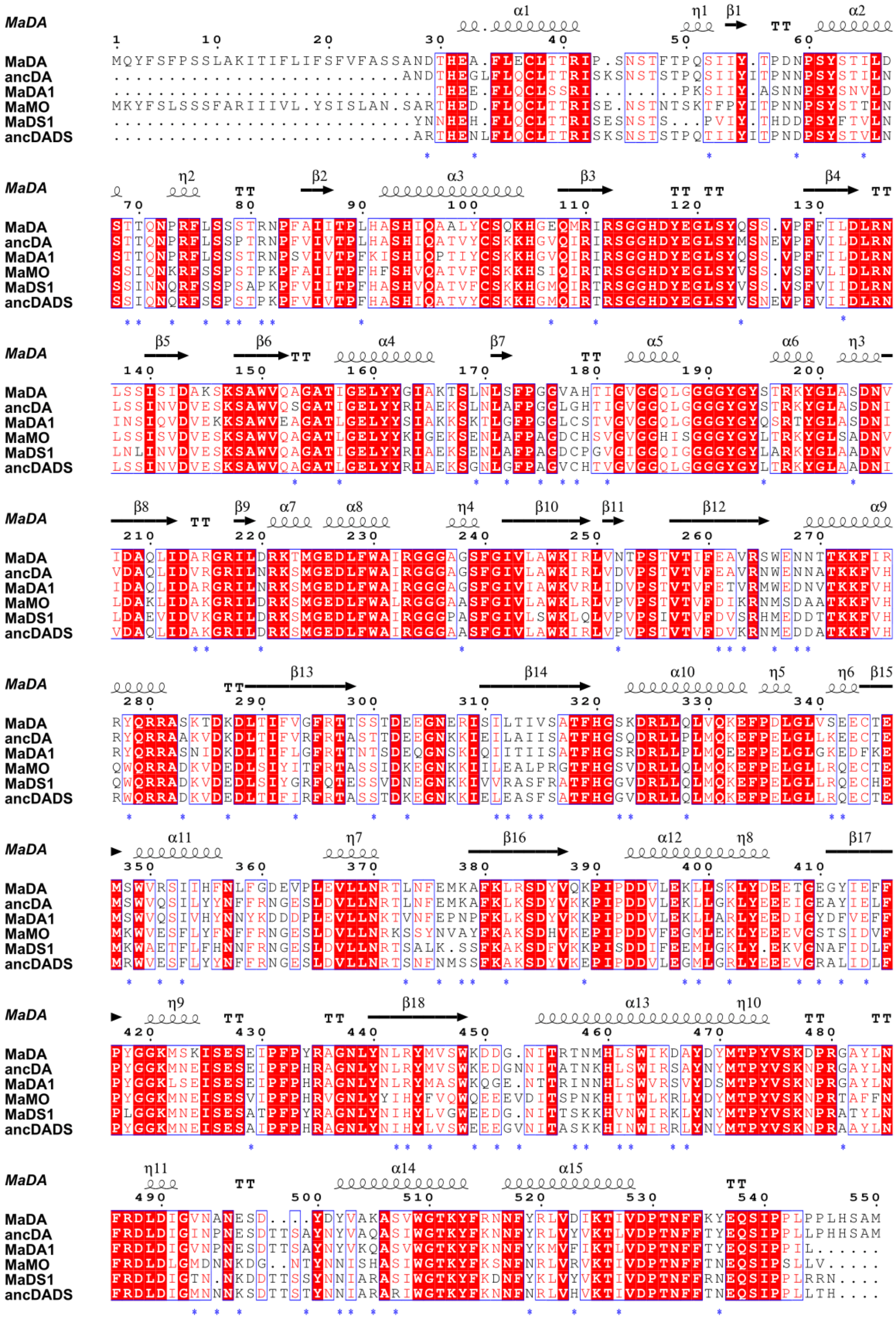

Supplementary Fig. 7| Sequence alignment of ancestral genes with extant genes.

Red filled boxes represent identical residues. Images were generated using ESPript 3.0. The 96 differential residues between ancDA and ancDADS are indicated by asterisks.

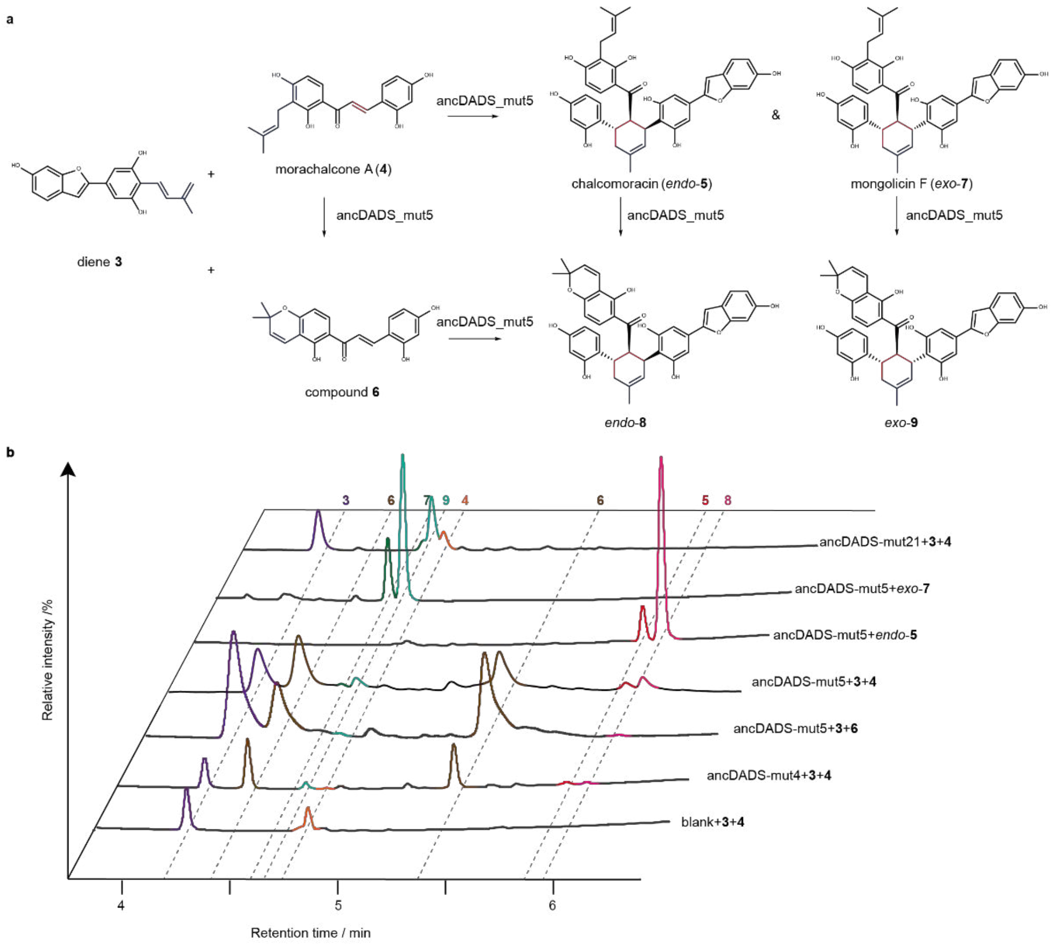

Supplementary Fig. 8| Occurrence of D-A activity.

a, The reaction scheme of ancDADS-mut4 (ancDADS-S348L-A357L-D389E-H418R). **b,** The UPLC analyses of enzymatic reactions catalysed by ancDADS-mut21, ancDADS-mut4 and ancDADS-mut5a. The reaction system comprised of 15 µg enzymes, 100 µM diene **3,** 100 µM dienophile **4** in a 100 µL Tris-HCl buffer (20 mM, pH 8.0). The reaction was performed at 50°C for 2 hours. The enzyme assay experiment was conducted with three independent replicates.

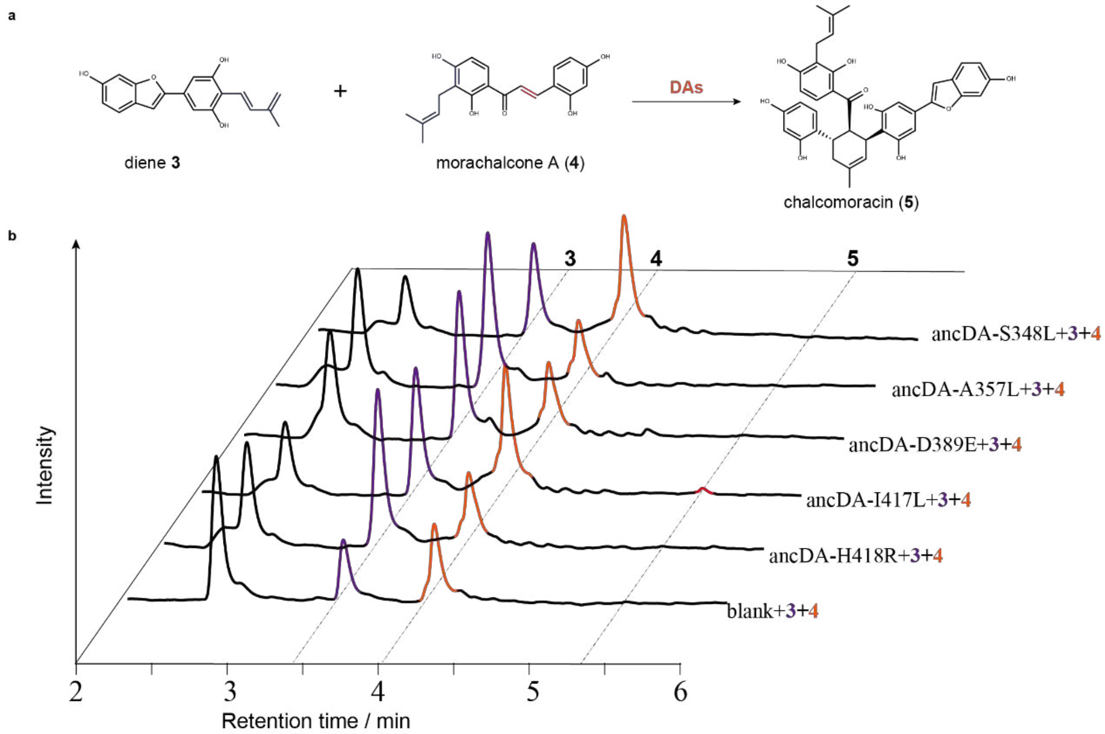

Supplementary Fig. 9| D-A activity of the ancDA mutants after prolonged reaction time.

a, The chemical formula for the enzymatic reaction of the DA assay. **b,** UPLC analysis of the enzyamtic assays catalysed by ancDA variants. The enzyme assays to detect D-A activity were carried out using 5 µg of ancDA variants, 1 µL of diene 3 (100 µM), 1 µL of morachalcone A (4) (100 µM), and a 20 mM Tris-HCl solution at pH 8.0. The mixture was incubated at 50°C for a longer time (2 hours) compared with the enzymatic assays in Fig.**3e** (7 minutes). The enzyme assay experiment was conducted with three independent replicates.

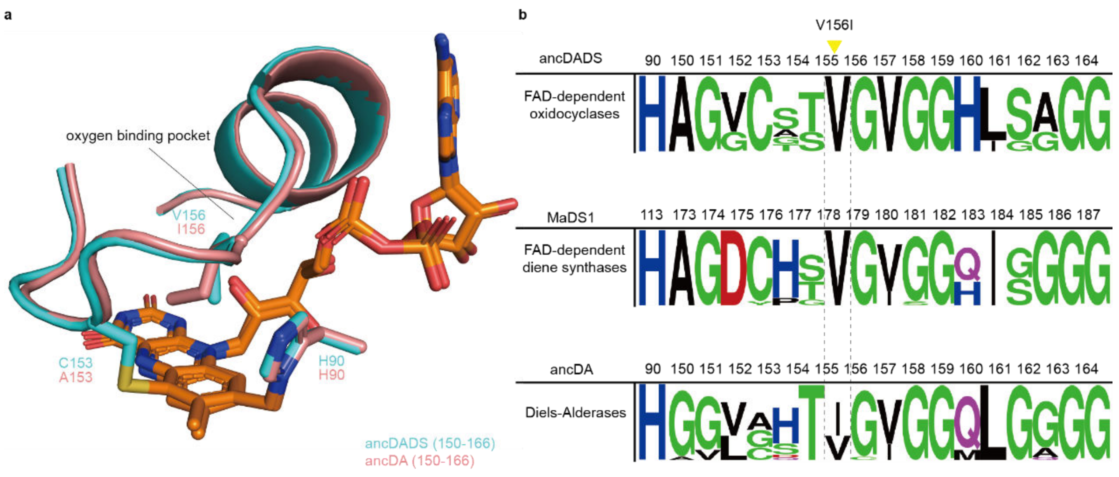

Supplementary Fig. 10| The conservation of the oxygen gatekeeper between DAs, OCs, and DSs.

a, The oxygen-binding pockets of ancDA and ancDADS. **b**, Conservation analysis results of residues on the oxygen-binding pocket among the three types of enzymes.

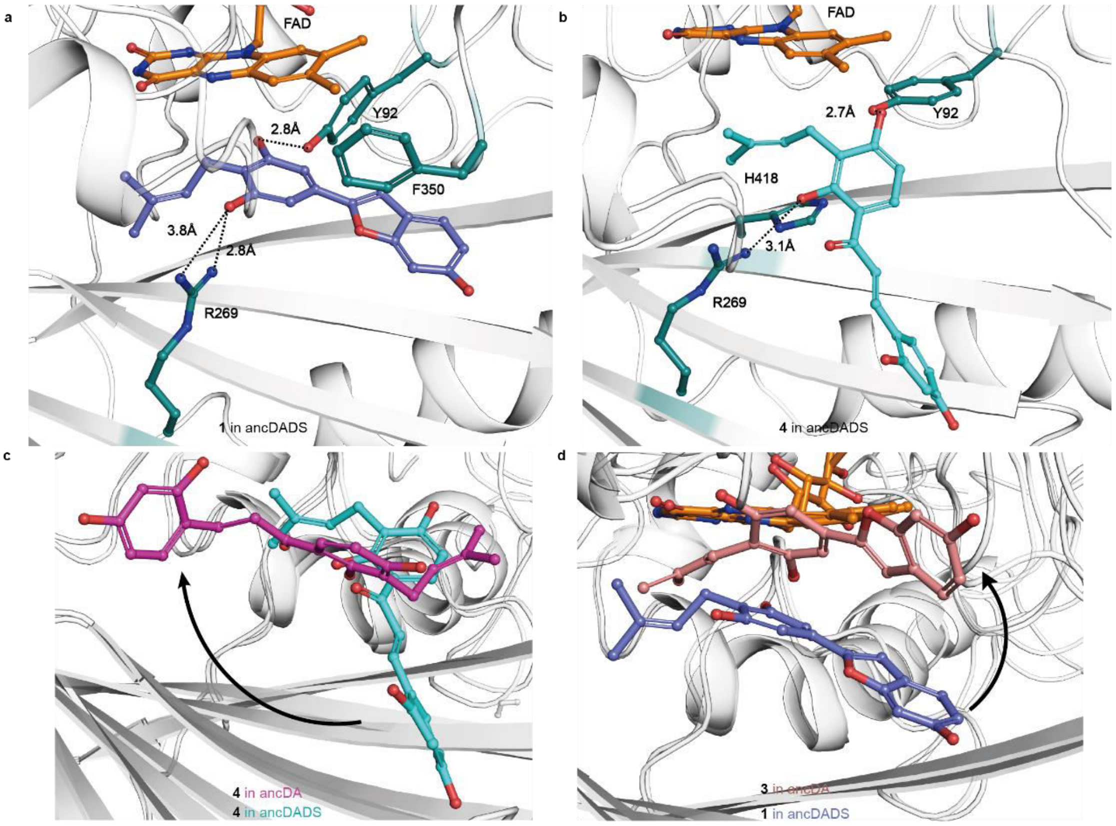

Supplementary Fig. 11| The binding mode change of substrates in the ancestor enzymes.

a, The binding patterns of moracin C (**1**) in ancDADS. **b**, The binding patterns of morachalcone A (**4**) in ancDADS. **c**, Superimposition of the binding pattern of morachalcone A (**4**) in ancDA and ancDADS. **d**, Superimposition of the binding pattern of diene **3** or moracin C (**1**) in ancDA and ancDADS.

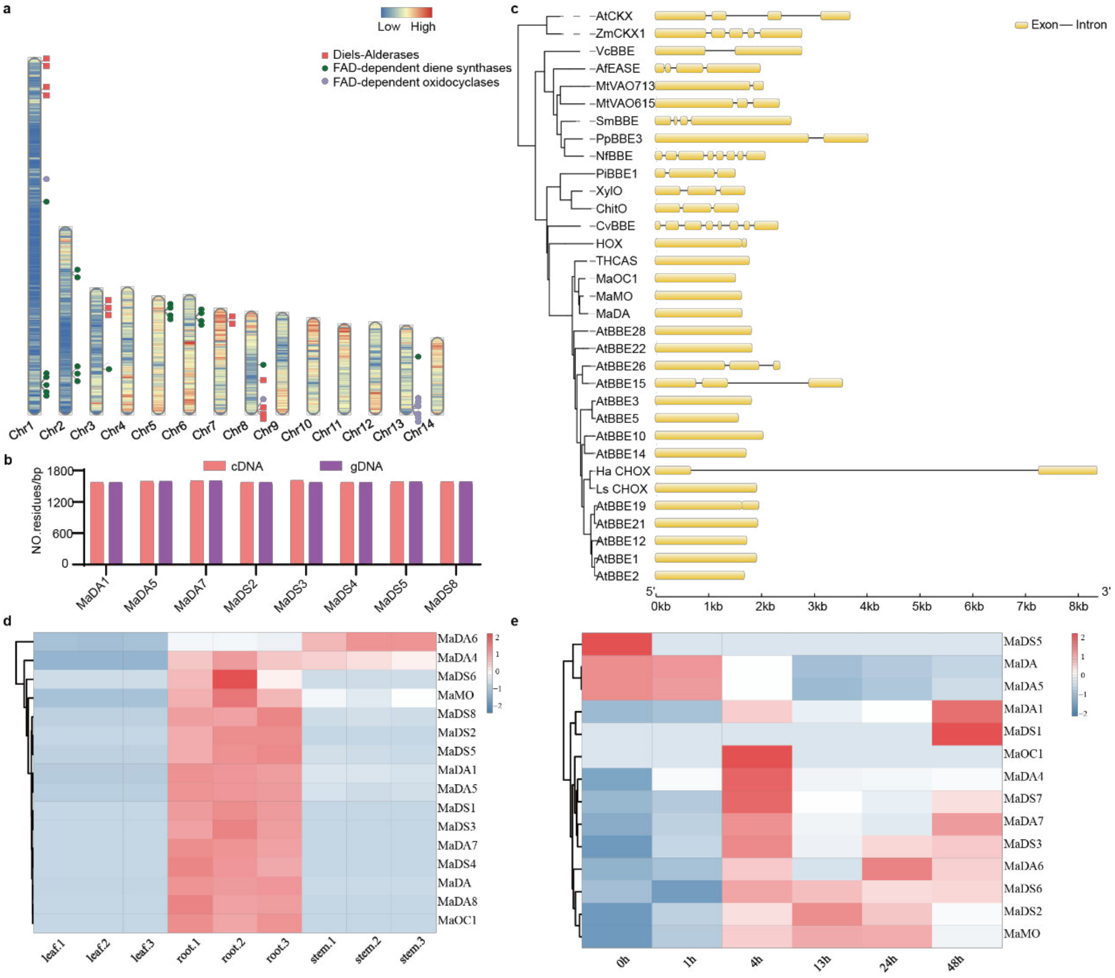

Supplementary Fig. 12| The genetic feature of DAs, DSs and OCs.

a, Chromosomal locations of DAs, DSs and OCs in *Morus alba.* A total of 13 DAs, 22 DSs and 8 OCs were mapped to all 14 chromosomes of *Morus alba*. Chromosome colours represent the magnitude of gene density. **b,** Gene length of DAs, DSs and OCs in *Morus alba.* **c**, The gene structure of BBE-like enzymes was analysed using .gff files. **d-e,** The expression pattern of DAs, DSs and OCs in different tissues and at different times after ultraviolet radiation. The expression values were calculated using Transcripts Per Million (TPM), with the scale representing normalized expression value (log_2TPM).

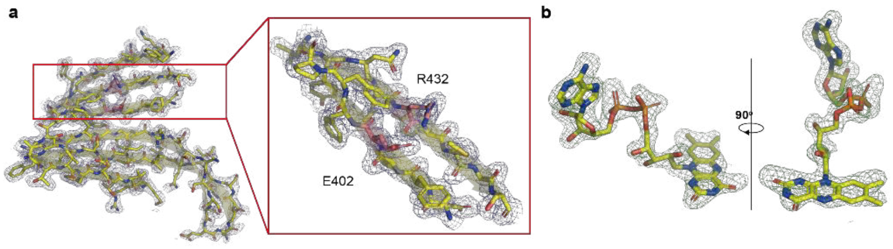

Supplementary Fig. 13| Density maps of crystal structure of MaDA1.

a, The electron density map for the six β-sheets (β12-14 and β16-18) substrate-binding pocket. The 2mFo-DFc difference electron density is displayed in blue mesh, contoured at 1.0σ. The amplified image of β17(400-406) and β18 (428-436) contains the important amino acids E402 and R432. **b,** Omit maps for FAD cofactor. The mFo-DFc difference electron density for FAD is displayed in green mesh, contoured at 3.5σ. The FAD is shown as stick model and colored by elements.

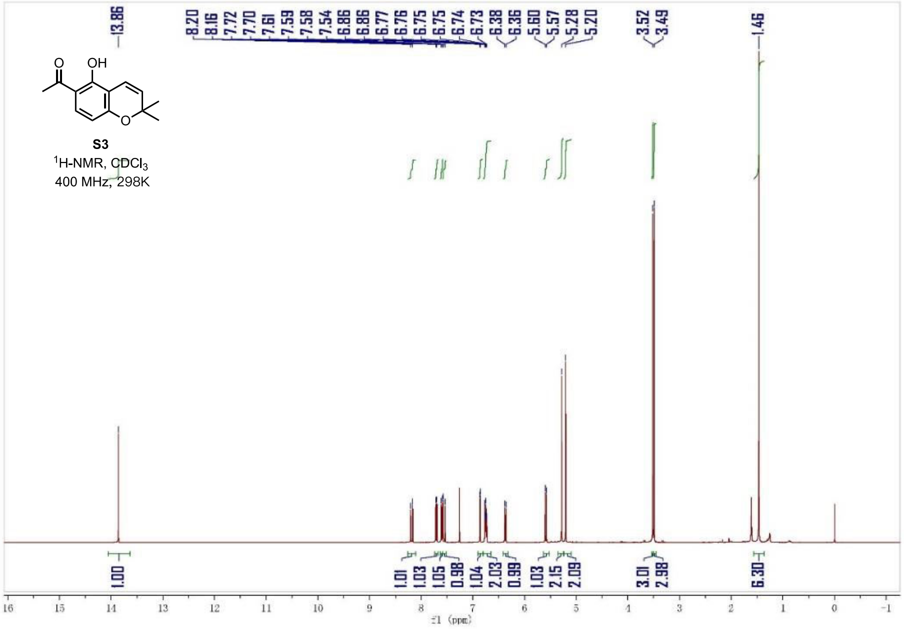

Supplementary Fig. 14| ^{1}H-NMR spectra of compound S3.

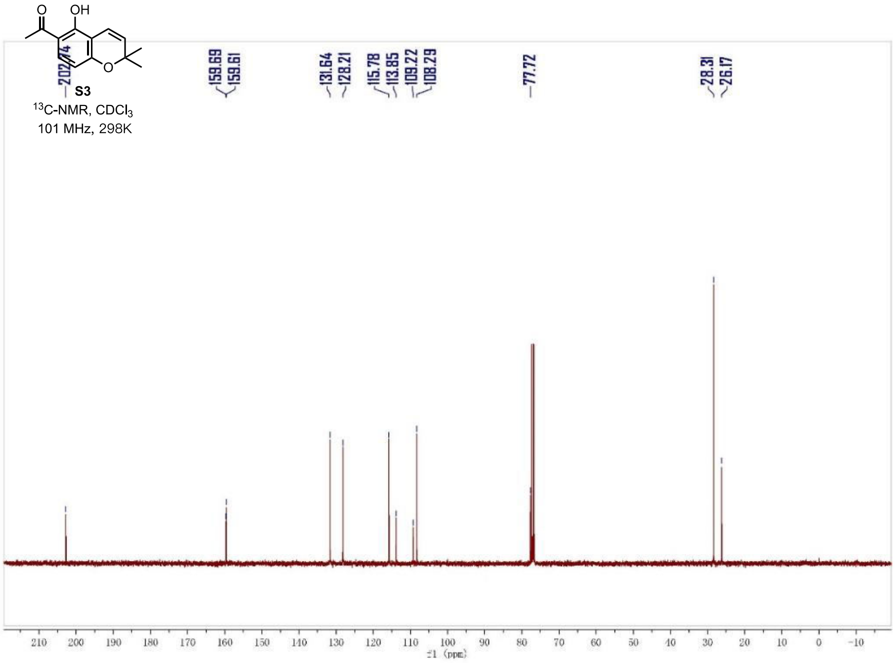

Supplementary Fig. 15| ^{13}C-NMR spectra of compound S3.

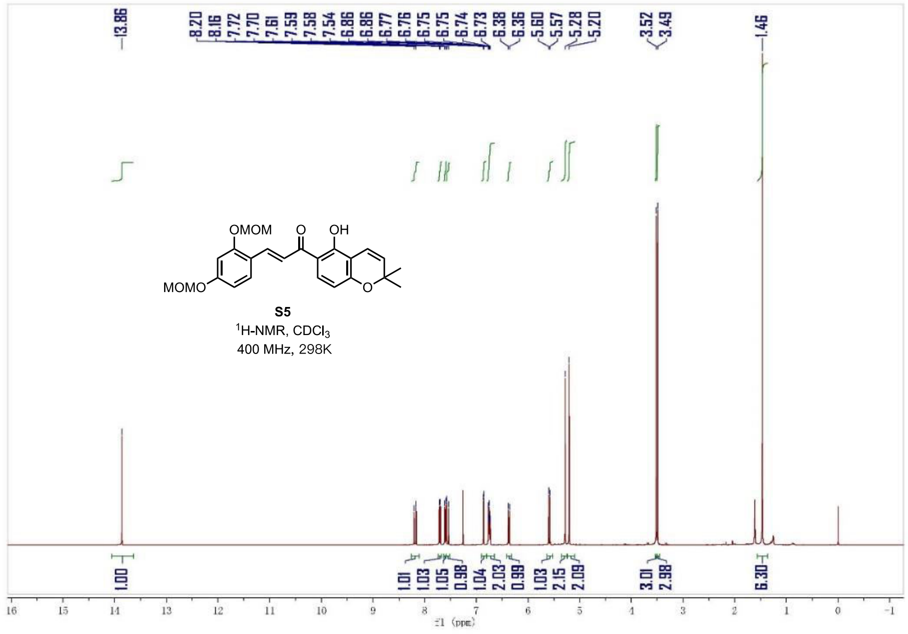

Supplementary Fig. 16| ^{1}H-NMR spectra of compound S5.

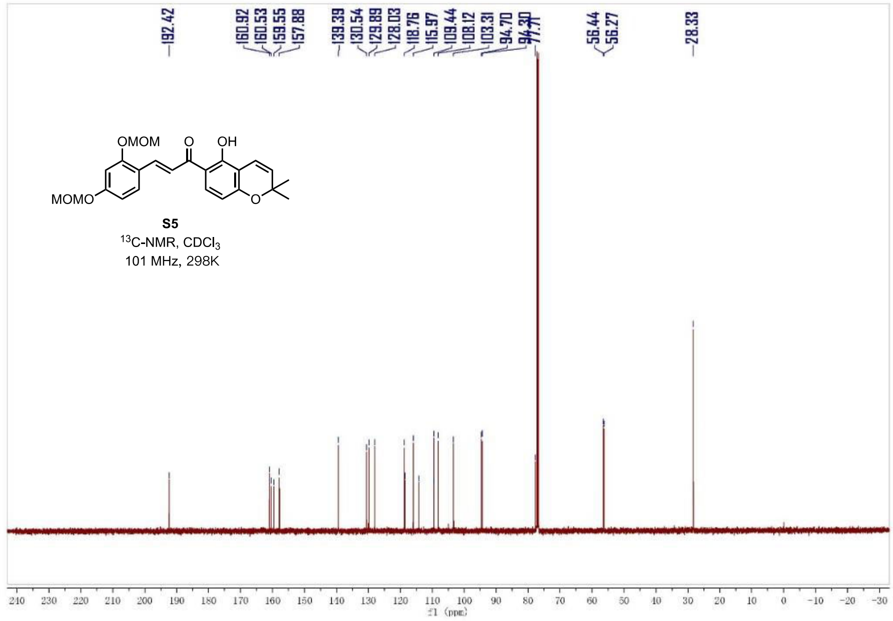

Supplementary Fig. 17| ^{13}C-NMR spectra of compound S5.

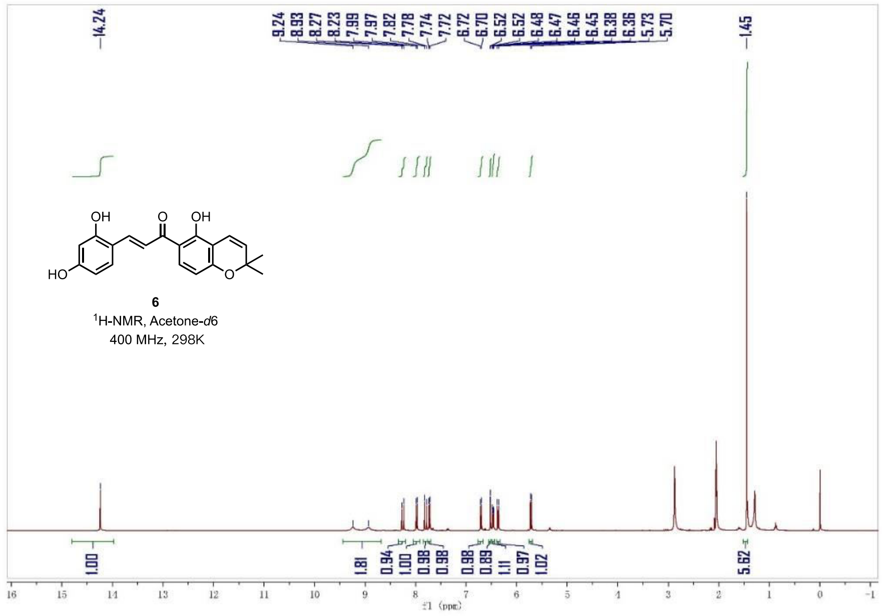

Supplementary Fig. 18| ^{1}H-NMR spectra of compound 6.

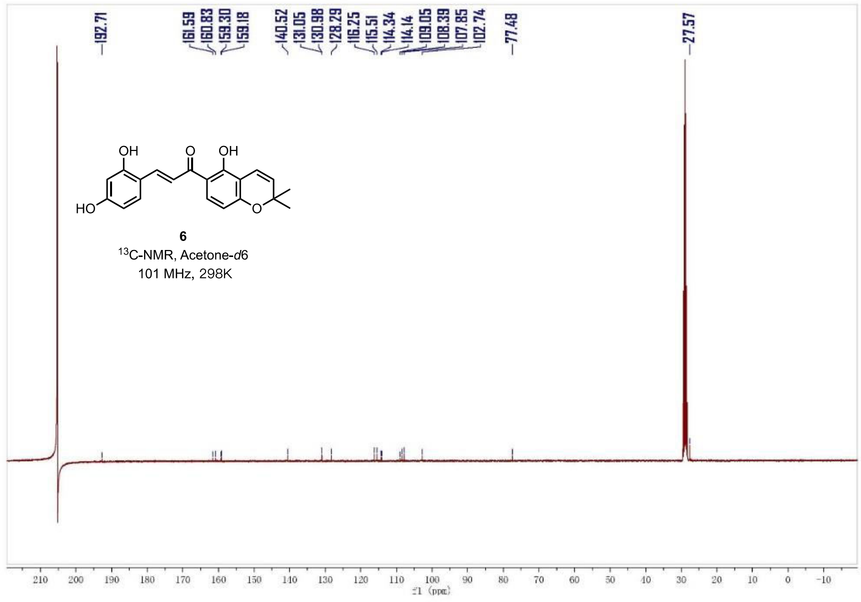

Supplementary Fig. 19| ^{13}C-NMR spectra of compound 6.

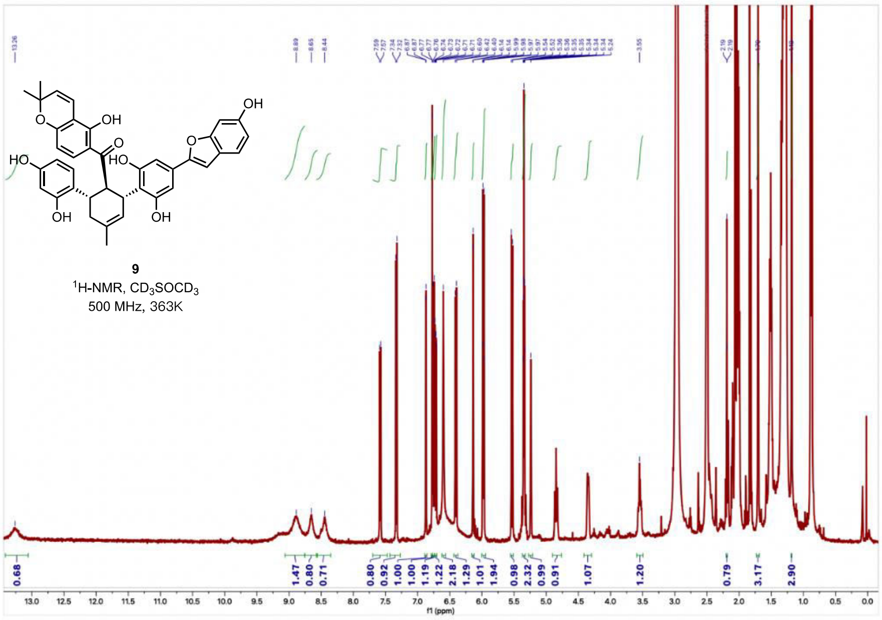

Supplementary Fig. 20| ^{1}H-NMR spectra of compound 9.

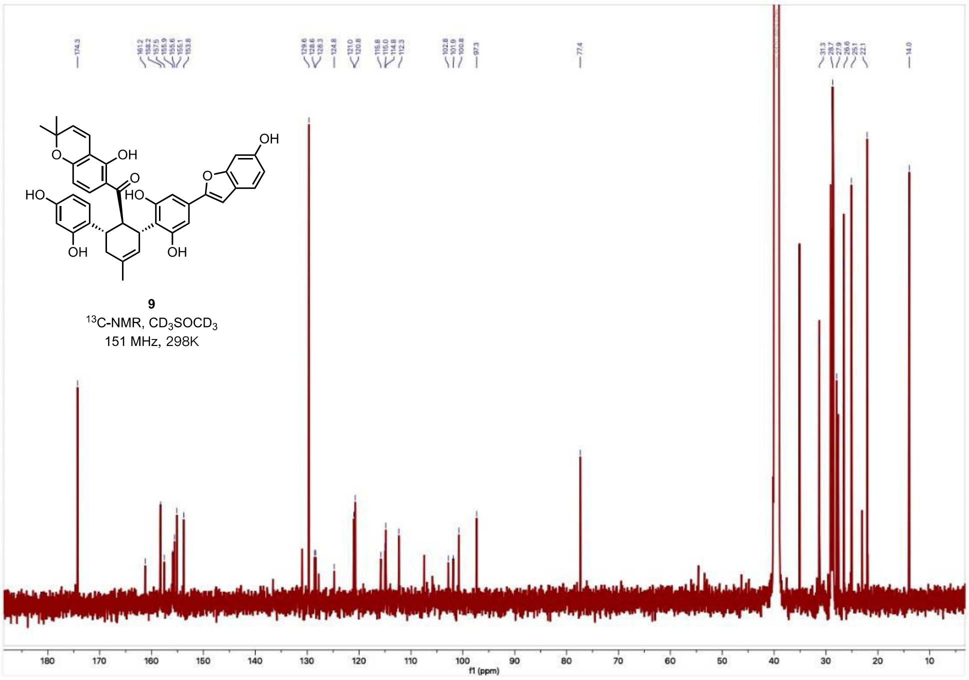

Supplementary Fig. 21| ^{13}C-NMR spectra of compound 9.

Supplementary Table 1. Information on DAs, DSs and OCs of Moraceae species.

For transcriptome data analysis, raw sequencing data was initially downloaded from NCBI. Then, the identification of the BBE-like enzyme families was carried out using HMMER after *de novo* assembly and annotation. Following this, phylogenetic trees, constructed using the maximum likelihood method, assisted in the prediction of DAs, DSs and OCs. Genomic data mining involved conducting a blast analysis directly on data retrieved from NCBI. BBE: BBE-like enzymes; DA: Diels-Alderases; DS: Diene Synthase; OC: oxidocyclases; NP: natural products.

Family	Species	abbr.	Run	proteins	BBE	DA	DS	OC	NP
Moraceae	*Artocarpus altilis*	Aal	SRR5997539	26952	1	0	0	0	Not yet
Moraceae	*Artocarpus mariannensis*	Ama	SRR5997531	29644	1	0	0	0	Not yet
Moraceae	*Artocarpus nanchuanensis*	Ana	SRR11623450	58347	53	0	15	0	Not yet
Moraceae	*Brosimum alicastrum*	Bal	SRR3953675	22313	5	0	8	0	Not yet
Moraceae	*Broussonetia papyrifera*	Bpa	SRR8363884	42034	7	0	1	0	Not yet
Moraceae	*Ficus altissima*	Fal	SRR14000707	46098	16	0	5	0	Not yet
Moraceae	*Ficus esquiroliana*	Fes	SRR7892351	30709	8	0	2	0	Not yet
Moraceae	*Ficus hirta*	Fhi	SRR7887226	34585	10	0	3	0	Not yet
Moraceae	*Ficus microcarpa*	Fmi	SRR8594168	24073	1	0	0	0	Not yet
Moraceae	*Ficus pumila*	Fpu	SRR6007382	34439	17	0	4	0	Not yet
Moraceae	*Ficus religiosa*	Fre	ERR2040415	22491	6	0	0	0	Not yet
Moraceae	*Ficus tikoua*	Fti	SRR6663247	39312	19	0	9	0	Not yet
Moraceae	*Morus indica*	Min	SRR8209721	20603	3	1	0	0	Not yet
Moraceae	*Morus macroura*	Mma	SRR3084265	48768	12	1	1	0	YES[1]
Moraceae	*Morus nigra*	Mni	ERR2040414	20603	4	1	0	0	YES[2]
Moraceae	*Morus serrata*	Mse	SRR3061576	48309	16	1	1	0	Not yet
			genome_tblastn						
Moraceae	*Artocarpus camansi*	Aca	-	-	70	0	6	1	Not yet
Moraceae	*Artocarpus heterophyllus*	Ahe	-	-	32	2	14	0	YES[3]
Moraceae	*Ficus carica*	Fca	-	-	34	0	22	17	Not yet
Moraceae	*Ficus erecta*	Fer	-	-	71	0	30	3	Not yet
Moraceae	*Morus alba*	Mal	-	27473	46	7	9	1	YES[4]
Moraceae	*Morus notabilis*	Mno	-	27648	37	6	9	5	YES[5]
Cannabaceae	*Cannabis sativa*	Csa	-	33674	54	0	0	0	Not yet
Cannabaceae	*Humulus lupulus*	Hlu	-	-	28	0	0	0	Not yet
Ulmaceae	*Ulmus americana*	Uam	-	-	14	0	0	0	Not yet
Urticaceae	*Boehmeria nivea*	Bni	-	-	64	0	0	0	Not yet

Supplementary Table 2. Primer information on DAs, DSs and OCs.

gene_ID	primer	primer sequence (5'→3')	size/bp	Tm/°C
MaOC1	forward	TATTTTCAGGGATCCCATGAACACTTTCTTCAATGCCTC	39	57
	reverse	CTTCTCGACAAGCTTTTAAGGGGCAAGAAGAGATGGAATG	40	58
MaDA1	forward	TATTTTCAGGGATCCAATCACACTCATGAAGAGTTTCTTC	40	58
	reverse	CTTCTCGACAAGCTTCTAGTACTTCTCGACAAGCTTATG	39	55
MaDA4	forward	TATTTTCAGGGATCCGATCAGATTGGCCATGAAGGC	36	58
	reverse	CTTCTCGACAAGCTTCTAGCGTTTATAATGCGGGCTCAG	39	58
MaDA5	forward	TATTTTCAGGGATCCTCCAACGACACTCATGAAGC	35	55
	reverse	CTTCTCGACAAGCTTCTAGTACTTCTCGACAAGCTTC	37	55
MaDA6	forward	TATTTTCAGGGATCCGATCAAATTGGTCATGAAGGCTTTC	40	57
	reverse	CTTCTCGACAAGCTTCTAGTACTTCTCGACAAGCTTCC	38	57
MaDA7	forward	TATTTTCAGGGATCCAACCACACTCATGATGGCTTTC	37	58
	reverse	CTTCTCGACAAGCTTCTAGTACTTCTCGACAAGCTTAAG	39	56
MaDA8	forward	TATTTTCAGGGATCCCATGAAAGCTTTCTTGAGTGCTTG	39	57
	reverse	CTTCTCGACAAGCTTCTAGTACTTCTCGACAAGCTTATG	39	56
MaDS1	forward	TATTTTCAGGGATCCTATAACAACCATGAACATTTTCTGCAG	42	56
	reverse	CTTCTCGACAAGCTTGTTGCGGCGCAGC	28	56
MaDS2	forward	TATTTTCAGGGATCCGCGCGCACTCATGAAGAC	33	58
	reverse	CTTCTCGACAAGCTTTTACACAAGAAGAGATGGAATGCTTTG	42	57
MaDS3	forward	TATTTTCAGGGATCCGCGCGCACTCATGAAGAC	33	58
	reverse	CTTCTCGACAAGCTTTTATGATCCTCTAGTACTTCTCGACAAG	43	57
MaDS4	forward	TATTTTCAGGGATCCGTGCCCACTCATGAAGACTTTC	37	57
	reverse	CTTCTCGACAAGCTTTTACACAAAAAGAGATGGGATGCTTTG	42	57
MaDS5	forward	TATTTTCAGGGATCCGATTTGAAACACACTCATGAAGGC	39	57
	reverse	CTTCTCGACAAGCTTTTAATGCGTAAGGATCGGTGGG	37	58
MaDS6	forward	TATTTTCAGGGATCCTATCACAATCATGAAGACTTTCTTCAATG	44	55
	reverse	CTTCTCGACAAGCTTTCAATGAAGAGGTGGGATGC	35	56
MaDS7	forward	TATTTTCAGGGATCCTATCACAATCATGAAAACTTTCTTCAATG	44	54
	reverse	CTTCTCGACAAGCTTCTAGTACTTCTCGACAAGCTTTTTC	40	56
ancDA	forward	TATTTTCAGGGATCCCACGAGGGTCTGTTCCTGC	34	59
	reverse	CTTCTCGACAAGCTTGTGTGGCAGCAGAGGAGG	33	59
ancDADS	forward	TATTTTCAGGGATCCCACGAAAACCTGTTCCTGCAG	36	59
	reverse	CTTCTCGACAAGCTTGTGAGTCAACAGGGGTGGG	34	59

Supplementary Table 3. Data collection and refinement statistics of MaDA1.

	MaDA1
**Data collection**	
Space group	$P2_12_12_1$
Cell dimensions	
a, *b*, *c* (Å)	82.78, 104.52, 141.65
α, β, γ (°)	90,90,90
Resolution (Å)	20.0-2.10(2.10-2.14)
R_{meas}	0.410(3.257)
I / σI	4.7(4.76)
Completeness (%)	99.8(96.3)
Redundancy	12.7(12.3)
**Refinement**	
Resolution (Å)	20.0-2.10(2.05-2.00)
No. reflections	69262
R_{work} / R_{free}	0.191/0.225
No. atoms	
Protein	8011
Ligand/ion	148
Water	594
B-factors	
Protein	25.18
Ligand/ion	22.11
Water	32.71
R.m.s. deviations	
Bond lengths (Å)	0.007
Bond angles (°)	0.836

*One crystal was used for each structure. *Values in parentheses are for highest-resolution shell.

Supplementary References

1. Dai, S.-J. et al. New Diels-Alder Type Adducts from *Morus macroura* and Their Anti-oxidant Activities. *Chem. Pharm. Bull.* **52**, 1190-1193 (2004).
2. Ferrari, F., Monacelli, B. & Messana, I. Comparison Between in Vivo and in Vitro Metabolite Production of *Morus nigra*. *Planta Med.* **65**, 85-87 (1999).
3. Hano, Y., Aida, M. & Nomura, T. Two new natural diels alder type adducts from the root bark of *Artocarpus heterophyllus*. *J. Nat. Prod.* **53**, 391-395 (1990).
4. Nomura, T. & Hano, Y. Isoprenoid-substituted phenolic compounds of moraceous plants. *Nat. Prod. Rep.* **11**, 205-218 (1994).
5. Wang, M. et al. Diels-Alder adducts with PTP1B inhibition from *Morus notabilis*. *Phytochemistry* **109**, 140-146 (2015).
6. Hano, Y.; Kohno, H.; Itoh, M.; Nomura, T., Structures of Three New 2-Arylbenzofuran Derivatives from the Chinese Crude Drug "Sang-Bai-Pi" (Morus Root Bark). *Chem. Pharm. Bull.* **33**, 5294-5300(1985).
