# Supplementary material for: The evolutionary origin of naturally occurring intermolecular Diels-Alderases from Morus alba
Source: Nat Commun. 2024 Mar 20;15:2492. doi: 10.1038/s41467-024-46845-0 (PMC10954736; doi:10.1038/s41467-024-46845-0)
Supplement: Supplementary file 3 — Reporting Summary [file 41467_2024_46845_MOESM3_ESM.pdf]

nature portfolio

Corresponding author(s): Lei Gao, Xiaoguang Lei

Last updated by author(s): Feb 25, 2024

Reporting Summary

Nature Portfolio wishes to improve the reproducibility of the work that we publish. This form provides structure for consistency and transparency in reporting. For further information on Nature Portfolio policies, see our Editorial Policies and the Editorial Policy Checklist.

Statistics

For all statistical analyses, confirm that the following items are present in the figure legend, table legend, main text, or Methods section.

n/a	Confirmed	
☐	☒	The exact sample size (*n*) for each experimental group/condition, given as a discrete number and unit of measurement
☐	☒	A statement on whether measurements were taken from distinct samples or whether the same sample was measured repeatedly
☒	☐	The statistical test(s) used AND whether they are one- or two-sided<br>*Only common tests should be described solely by name; describe more complex techniques in the Methods section.*
☒	☐	A description of all covariates tested
☒	☐	A description of any assumptions or corrections, such as tests of normality and adjustment for multiple comparisons
☐	☒	A full description of the statistical parameters including central tendency (e.g. means) or other basic estimates (e.g. regression coefficient) AND variation (e.g. standard deviation) or associated estimates of uncertainty (e.g. confidence intervals)
☒	☐	For null hypothesis testing, the test statistic (e.g. *F*, *t*, *r*) with confidence intervals, effect sizes, degrees of freedom and *P* value noted<br>*Give P values as exact values whenever suitable.*
☒	☐	For Bayesian analysis, information on the choice of priors and Markov chain Monte Carlo settings
☒	☐	For hierarchical and complex designs, identification of the appropriate level for tests and full reporting of outcomes
☒	☐	Estimates of effect sizes (e.g. Cohen's *d*, Pearson's *r*), indicating how they were calculated

Our web collection on statistics for biologists contains articles on many of the points above.

Software and code

Policy information about availability of computer code

Data collection	GenBank; XDS
Data analysis	HMMER v3.3.2; Trinity; Transdecoder V5.5.0; FastQC; TGI Clustering tools (TGICL); MAFFT v7; CD-HIT; Gblock; DAMBE v5; IQTREE v1.6.12; MrBayes 3.2.7a; PAML v4; PyMol 2.07; Interactive Tree Of Life (iTOL) software (https://itol.embl.de/); R Programming language (4.3.1); POVME 3.0; MScanX; The Gene Structure Display Server 2.0; Trimmomatic v0.39;Kallisto; ChemDraw 21.0.0; Phaser:(autobuild, refine, phaser) 1.18.2-3874; Coot: 0.8.9.2 EL;Adobe Illustrator 22.1;Schrodinger Maestro 13.1; AlphaFold v2.2.0; GraphPad Prism 8

For manuscripts utilizing custom algorithms or software that are central to the research but not yet described in published literature, software must be made available to editors and reviewers. We strongly encourage code deposition in a community repository (e.g. GitHub). See the Nature Portfolio guidelines for submitting code & software for further information.

Data

Policy information about availability of data

All manuscripts must include a data availability statement. This statement should provide the following information, where applicable:

- Accession codes, unique identifiers, or web links for publicly available datasets
- A description of any restrictions on data availability
- For clinical datasets or third party data, please ensure that the statement adheres to our policy

The data that support the findings of this study are available with this article and its Supplementary Information or are available from the corresponding authors upon request. The origins of the raw sequencing data analyzed in this study are elaborated in Supplementary Table 1. The gene sequences of MaDA5-8, MaDS1-7,

and MaOC1 are deposited in GenBank under accession NO. ON745422,ON787914-ON787924. The structural factors and coordinates of MaDA1 are deposited in the Protein Data Bank under ID 7YAV.

Research involving human participants, their data, or biological material

Policy information about studies with human participants or human data. See also policy information about sex, gender (identity/presentation), and sexual orientation and race, ethnicity and racism.

Reporting on sex and gender	N/A
Reporting on race, ethnicity, or other socially relevant groupings	N/A
Population characteristics	N/A
Recruitment	N/A
Ethics oversight	N/A

Note that full information on the approval of the study protocol must also be provided in the manuscript.

Field-specific reporting

Please select the one below that is the best fit for your research. If you are not sure, read the appropriate sections before making your selection.

[x] Life sciences   [ ] Behavioural & social sciences   [ ] Ecological, evolutionary & environmental sciences

For a reference copy of the document with all sections, see nature.com/documents/nr-reporting-summary-flat.pdf

Life sciences study design

All studies must disclose on these points even when the disclosure is negative.

Sample size	This study did not include a sample size calculation. A sample size of n=3 was chosen to conduct three biologically independent experiments in order to assess the activities of both wild-type enzymes and their mutants. These sample sizes are sufficient for the biochemical studies in this work.
Data exclusions	No data were excluded from this study.
Replication	The experiments were independently replicated and consistently yielded similar results. The number of replicates is specified in the figure legends.
Randomization	No randomization was required because genetically and biochemically identical samples were used.
Blinding	No blinding was required because biochemical experiments were performed in this work by individual researchers.

Reporting for specific materials, systems and methods

We require information from authors about some types of materials, experimental systems and methods used in many studies. Here, indicate whether each material, system or method listed is relevant to your study. If you are not sure if a list item applies to your research, read the appropriate section before selecting a response.

Materials & experimental systems

n/a	Involved in the study	
[x]	[ ]	Antibodies
[ ]	[x]	Eukaryotic cell lines
[x]	[ ]	Palaeontology and archaeology
[x]	[ ]	Animals and other organisms
[x]	[ ]	Clinical data
[x]	[ ]	Dual use research of concern
[x]	[ ]	Plants

Methods

n/a	Involved in the study	
[x]	[ ]	ChIP-seq
[x]	[ ]	Flow cytometry
[x]	[ ]	MRI-based neuroimaging

Eukaryotic cell lines

Policy information about cell lines and Sex and Gender in Research

Cell line source(s)	Sf9 insect cells (Invitrogen, USA).
Authentication	Cell lines were not authenticated.
Mycoplasma contamination	All cell lines were negative for mycoplasma contamination.
Commonly misidentified lines (See ICLAC register)	This study did not utilize commonly misidentified cell lines.

Plants

Seed stocks	Morus alba leaves were collected from Peking University, Beijing, China.
Novel plant genotypes	No new plant phenotypes have been identified.
Authentication	none
